# Synthesis and singlet oxygen generation of boron-2-(4,5-dibromo-1H-imidazole-2-yl)-3,5-dipyrazolopyridine complex for antimicrobial photodynamic therapy

**DOI:** 10.55730/1300-0527.3627

**Published:** 2023-10-11

**Authors:** Zeynep EKMEKÇİ, Seda KARAOĞLU

**Affiliations:** Department of Biomedical Engineering, Faculty of Technology, Isparta University of Applied Sciences, Isparta, Turkiye

**Keywords:** Antimicrobial photodynamic therapy, pyrazole, BOPIM, singlet oxygen

## Abstract

In this study, the pyridine side of a boron 2-(2′-pyridyl) imidazole (BOPIM) core, which has very few derivatives synthesized in the literature and can show fluorescence properties in solid form, was derivatized with 1-methylpyrazole, predicted to have activity against fungi or bacteria according to the literature. Additionally, its imidazole side was brominated to increase the efficiency of singlet oxygen production by increasing the intersystem crossing. The photophysical properties of the new synthesized BOPIM derivative were investigated in general organic solvents with different polarities. While the wavelength of the maximum absorbance was determined as 406 nm in CH_2_Cl_2_ and THF, the wavelength of the highest emission was measured at 497 nm in CH_3_CN solvent. The largest Stokes shift was determined as 104 nm in CH_3_CN. This value was considerably higher than those of many photosensitizers. The singlet oxygen generation potential of the BOPIM derivative was revealed using a 440-nm LED lamp in the presence of singlet oxygen scavenger 1,3-diphenylisobenzofuran (DPBF). Additionally, it was demonstrated that the BOPIM derivative had no toxic effects by measurements made in the dark.

## 1. Introduction

Photodynamic therapy (PDT) is known as a noninvasive treatment method that causes irreversible cell damage by reactive oxygen species formed because of excitation of a photosensitive molecule with appropriate light [1]. The number of people suffering from different types of cancer and infectious diseases is increasing. If the increase in cancer-related death rates continues in this way, it is estimated that it will reach 13.1 million in 2030 [2]. For this reason, the search for new drugs and methods for cancer treatment continues unabated. Compared to other treatment methods, PDT appears to have some advantages. Among them, it has a wide spectrum of action, does not create situations such as drug resistance, yields fast results, and has high selectivity, practicality, and low cost of application [3,4]. Due to these advantageous properties of PDT, research on photosensitizers, which are critical components of PDT, continues [2,5–8]. It has been stated that PDT has a particularly wide range of uses in the antimicrobial spectrum against bacteria, viruses, protozoa, and fungi [9,10]. If microbial cells are destroyed in PDT, the treatment can be called antimicrobial photodynamic therapy (aPDT) [1]. It is seen in the literature that some kinds of pyrazole molecules have antifungal effects. For example, due to the structural properties of succinate dehydrogenase inhibitors, several pyrazole-4-acetohydrazide derivatives potentially targeting fungal succinate dehydrogenase were synthesized and investigated for their antifungal effects against *Rhizoctonia solani*, *Fusarium graminearum*, and *Botrytis cinerea*. The effects of pyrazole-4-acetohydrazides against fungi were investigated and their effectiveness was demonstrated [11]. Hua et al. synthesized a series of pyrazole amide and waltherione alkaloid-derived pyrazole ester derivatives based on the positive results obtained from their previous studies. In vitro and in vivo fungicidal activities of the aforementioned synthesized compounds were investigated. It was found that one of the compounds revealed a good inhibition rate against *Physalospora piricola*. Additionally, the results were supported by observations made under transmission electron and fluorescence microscopes [12]. Santos et al. synthesized porphyrin and chlorine-containing pyrazole molecules based on the antibacterial, antifungal, and antiinflammatory effects of pyrazole derivatives. The aPDT activities of these molecules against planktonic and biofilm forms of *Escherichia coli*, a gram-negative bacterium, were investigated. The results showed that the pyrazole-containing porphyrin and chlorine compounds exhibited high aPDT activity against planktonic and biofilm forms of *E. coli* [13]. In the present study, a 2-(2′-pyridyl) imidazole (BOPIM) core, as one of the new photosensitizers in the literature [14,15], was used. The BOPIM structure, which has advantageous properties such as high Stokes shift, high quantum yield, fluorescence properties in solid state, and solubility in many solvents, was derivatized with 1-methyl pyrazole, which could have an antifungal effect. In addition to examining its photophysical properties, essential measurements were performed for its singlet oxygen generation potential, which is necessary for aPDT to be effective. According to the results, it was concluded that the new BOPIM derivative could be a candidate photosensitive molecule for aPDT.

## 2. Materials and methods

All chemical molecules used in synthesis and measurement were purchased from Sigma-Aldrich (St. Louis, MO, USA) and Acros (Waltham, MA, USA). ^1^H NMR and ^13^C NMR spectral measurements were performed with a Bruker instrument (Billerica, MA, USA) in the Chemistry Department of Middle East Technical University (Ankara, Türkiye). The mass spectra of all molecules synthesized for the first time were determined using a 1200/6210 Accurate-Mass TOF LC/MS (Agilent Technologies, Santa Clara, CA, USA) at the National Nanotechnology Research Center (Bilkent University, Ankara, Türkiye). Agilent Cary 60 and Agilent Cary Eclipse spectrophotometers (Agilent Technologies) were used in our research laboratory for absorption and fluorescence measurements. The Testboy TV335 device (ELBRO AG, Bülach, Switzerland) was used as a digital LED luxmeter to evaluate the intensity of the LED.

The synthesis pathway of the target BOPIM (**6**) is shown in the Scheme.

### 2.1. Synthesis of 3,5-bis(1-methyl-1H-pyrazole-4-yl) picolinealdehyde (3)

Synthesis of molecule **3** was performed in a procedure similar to that presented in the literature [16,17]. In this synthesis, 1-methylpyrazole-4-boronic acid pinacol ester (**1**) (197 mg, 0.095 mmol), 3,5-dibromopyridine-2-carboxaldehyde (**2**) (100.1 mg, 0.378 mmol), and PdCl_2_(PPh_3_)_2_ (26.5 mg, 0.038 mmol) were used. At the end of the synthesis of **3**, in the purification part of the procedure, the main product was first purified by column chromatography prepared with ethyl acetate. The column was then converted to a mixture of ethyl acetate:methanol (97:3, v/v) to obtain a white solid (**3**) (51.8 mg, 51%). ^1^H NMR (400 MHz, CDCl_3_): 10.20 (s, 1H), 8.85 (d, *J* = 2.0 Hz, 1H), 7.91 (s, 1H), 7.78–7.81 (m, 3H), 7.75 (s, 1H), 4.03 (s, 3H), 4.02 (s, 3H). ^13^C NMR (100 MHz, CDCl_3_): 191.2, 146.3, 145.2, 139.4, 137.2, 133.9, 131.8, 131.3, 130.9, 127.9, 118.3, 116.8, 39.2, 39.1. HRMS: Calcd. for C_14_H_13_N_5_O [M+H]^+^ 268.11929; found 268.11801, Δ = 4.76 ppm.

### 2.2. Synthesis of 2-(1H-imidazole-2-yl)-3,5-bis(1-methyl-1H-pyrazole-4-yl) pyridine (4)

Synthesis of molecule **4** was performed in a procedure similar to that presented in the literature [18]. In this synthesis, 3,5-bis(1-methyl-1*H*-pyrazole-4-yl) picoline aldehyde (**3**) (68 mg, 0.67 mmol), 7 mL EtOH, 40% aqueous glyoxal (88.6 μL, 0.78 mmol), and 25% NH_3_ (218.42 μL, 2.91 mmol) were used. At the end of the synthesis of **4**, a dark yellow solid mixture was obtained by evaporation of ethanol from the reaction mixture. This mixture was washed by adding small amounts of ethyl acetate. After washing, the remaining solid part was dissolved in ethyl acetate together with a small amount of methanol by heating. It was then allowed to crystallize under hexane vapor. The molecule (**4**), which passed into the solvent during the washing process, was purified by neutral column chromatography prepared with ethyl acetate. The molecule (**4**) was obtained in solid form with a very light yellow color from crystallization and column chromatography was combined (30.2 mg, 17%). ^1^H NMR (400 MHz, d-MeOH): 8.72 (dd, *J* = 5.2, *J* = 2.0 Hz, 1H), 8.20 (d, *J* = 5.2 Hz, 1H), 8.15 (dd, *J* = 6.4, *J* = 2.0 Hz, 1H), 8.03 (d, *J* = 4.8 Hz, 1H), 7.52 (d, *J* = 4.0 Hz, 1H), 7.27 (d, *J* = 4.0 Hz, 1H), 7.18 (d, *J* = 2.8 Hz, 2H), 3.98 (d, *J* = 3.6 Hz, 3H), 3.87 (d, *J* = 3.6 Hz, 3H). ^13^C NMR (100 MHz, d-MeOH): 145.0, 143.6, 143.2, 137.8, 136.4, 132.9, 129.9, 129.3, 128.8, 128.5, 118.6, 118.4, 37.6, 37.4 (14 signals were observed instead of 16 signals). HRMS: Calcd. for C_16_H_15_N_7_ [M+H]^+^ 306.14617; found 306.14625, Δ = −1.08 ppm.

### 2.3. Synthesis of 2-(4,5-dibromo-1*H*-imidazol-2-yl)-3,5-bis(1-methyl-1*H*-pyrazole-4-yl) pyridine (5)

Synthesis of molecule **5** was performed in a procedure similar to that presented in the literature [18–20]. In this synthesis, molecule **4** (30.2 mg, 0.1 mmol), 4.5 mL of CHCl_3_, and Br_2_ (31.82 mg, 10.2 μL, 0.2 mmol) dissolved in 1 mL of CHCl_3_ were used. At the end of the reaction, the solvent in the environment was removed. The compound (**5**) was purified by column chromatography with chloroform:methanol (98:2, v/v). The compound (**5**) was obtained in the form of a yellow solid (23 mg, 7%). ^1^H NMR (400 MHz, d-MeOH): 8.69 (s, 1H), 8.16 (s, 1H), 8.06–8.01 (m, 1H), 7.99 (s, 1H), 7.73 (s, 1H), 7.39 (s, 1H), 3.98 (s, 3H), 3.92 (s, 3H). ^13^C NMR (100 MHz, d-MeOH): 146.4, 143.4, 141.7, 138.1, 136.4, 133.6, 130.4, 129.5, 128.6, 128.3, 118.3, 118.1, 37.6, 37.5.14 (out of 16 expected signals). HRMS: Calcd. for C_16_H_13_Br_2_N_7_ [M+H]^+^ 461.9672; found 461.96590, Δ = 2.8 ppm.

### 2.4. Synthesis of boron-2-(4,5-dibromo-1H-imidazole-2-yl)-3,5-dipyrazolopyridine complex (6)

Synthesis of molecule **6** was performed in a procedure similar to that presented in the literature [18–20]. In the synthesis, molecule **5** (43.6 mg, 0.094 mmol) in 2 mL of CHCl_3_ passed through nitrogen gas, 196 μL of Et_3_N (1.41 mmol), and 196 μL of BF_3_.OEt_2_ (1.56 mmol) were used. After the reaction, the mixture was purified by column chromatography with chloroform:methanol (94.6:5.4, v/v). A light green solid target molecule (**6**) with fluorescence properties in solid form was obtained (4 mg, 8%). ^1^H NMR (400 MHz, d-DMSO): 9.05 (s, 1H), 8.78 (s, 1H), 8.71 (d, *J* = 1.2 Hz, 1H), 8.59 (s, 1H), 8.40 (s, 1H), 8.29 (s, 1H), 3.91 (s, 3H), 3.86 (s, 3H). ^13^C NMR (100 MHz, d-DMSO): 147.4, 140.1, 139.1, 137.9, 137.1, 134.9, 132.9, 131.5, 130.7, 126.9, 120.6, 116.4, 114.7, 106.6 (two C peaks of methyl groups at approximately 40 ppm were under the C peaks that belonged to d-DMSO). HRMS: Calcd. for C_16_H_12_BBr_2_F_2_N_7_ [M+Cl]^−^ 543.92761; found 542.93142, Δ = −0.33 ppm.

All NMR and HRMS spectra are provided in the Supplementary Information.

## 3. Results and discussion

**Figure 1:**
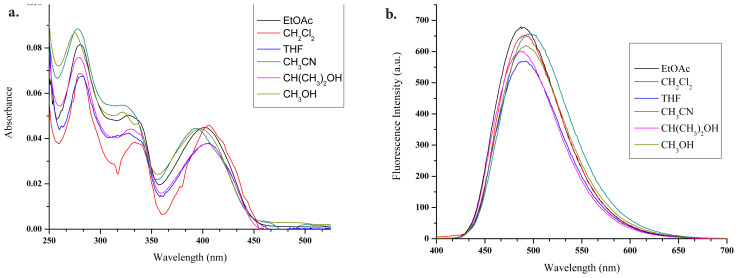
Spectra of BOPIM (**6**) (25 μM) in different solvents (EtOAc, CH_2_Cl_2_, THF, CH_3_CN, CH(CH_3_)_2_OH, and CH_3_OH): a) absorbance, b) fluorescence. Excitation: 385 nm, PMT voltage: 600 V.

The absorbance and emission values of target molecule **6** were measured in general organic solvents with different polarities (EtOAc, CH_2_Cl_2_, THF, CH_3_CN, CH(CH_3_)_2_OH, and CH_3_OH) to reveal its solvent effects and the results are given in Figure 1.

The absorbance wavelength range of the BOPIM molecule (**6**) varies between 393 nm and 406 nm. The wavelength of the maximum absorbance was determined as 406 nm in CH_2_Cl_2_ and THF. The emission wavelength range varied between 487 nm and 497 nm, while the wavelength of the highest emission was measured at 497 nm in CH_3_CN solvent. The largest Stokes shift was determined as 104 nm in CH_3_CN. The highest quantum efficiency was calculated as 0.262 in THF (Table). Fluorescein (Φ_F_ = 0.95, 0.1 M NaOH) was used as a reference material in quantum yield calculations [21].

**Figure 2:**
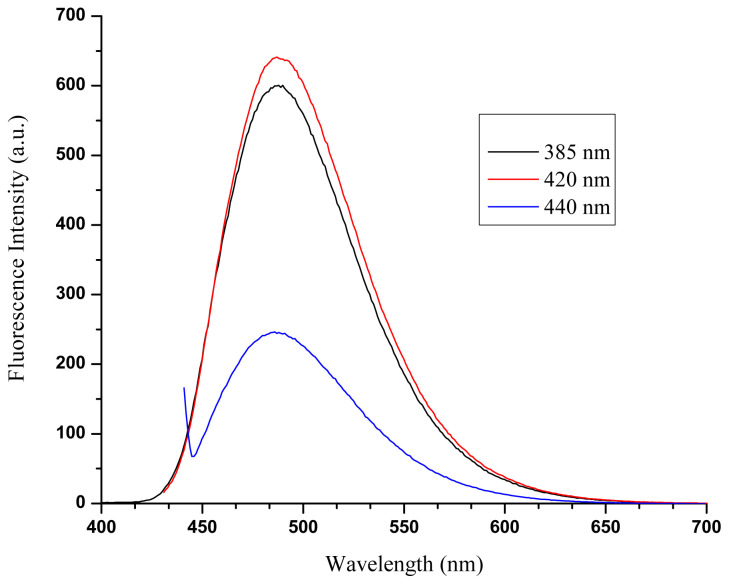
Emission spectra because of stimulation of BOPIM (**6**) at different wavelengths in isopropyl alcohol.

To understand the ability of the BOPIM (**6**) to produce singlet oxygen, 1,3-diphenylisobenzofuran (DPBF) with an absorption band at 410 nm, which is known as a singlet oxygen trap, was used. Since the absorbance values of molecule **6** and DPBF were close to each other, rapid degradation of DPBF was encountered at the wavelength required to excite the BOPIM (**6**). A similar situation was observed for another BOPIM derivative synthesized in a previous study conducted by our group [12]. Thus, a suitable wavelength at which the BOPIM (**6**) could be excited and the DPBF would not decay rapidly was sought. In this way, it could be said that the decrease in the absorbance of DPBF was also due to the singlet oxygen formed as a result of the excitation of BOPIM (**6**). The wavelength was determined as 440 nm in our study (Figure 2). A wavelength determination study was carried out using isopropyl alcohol, where measurements regarding singlet oxygen generation were performed.

**Figure 3:**
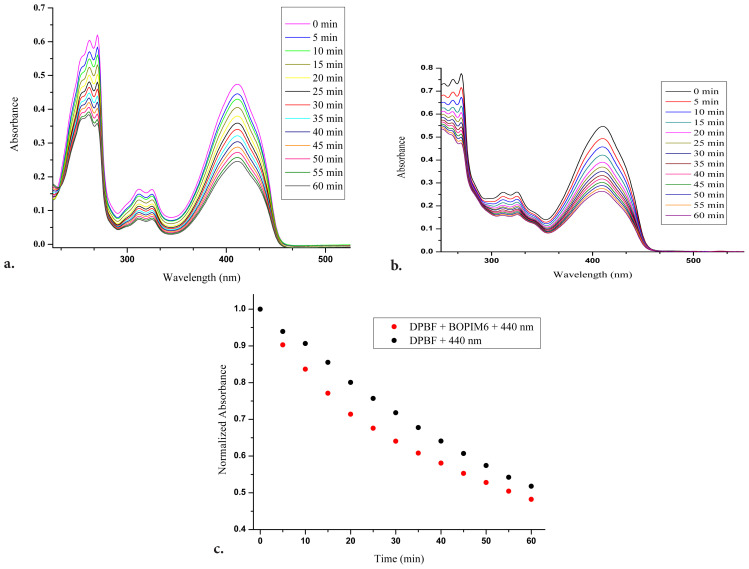
Absorbance spectra of a) DPBF (25 μM) under 440 nm light and b) the solution with DPBF (25 μM) and BOPIM complex (**6**) (50 μM) under 440 nm light. c) Graph of normalized values at 410 nm of the spectra given in a and b with respect to time.

At this wavelength, DPBF would not degrade rapidly and good emission intensity was observed for molecule **6**. The total appropriate concentrations were also adjusted so that the total absorbance value of the mixture of DPBF and the BOPIM derivative (**6**) did not exceed 1. Figure 3a shows the absorbance spectrum of DPBF (25 μM) in isopropyl alcohol measured by exposure to light with a wavelength of 440 nm at 5-min intervals. Figure 3b demonstrates the absorbance spectrum of the mixture in oxygenated isopropyl alcohol containing DPBF (25 μM) and BOPIM (**6**) (50 μM) measured at 5-min intervals under light with a wavelength of 440 nm. Its light intensity was 247.2 lx and 1.58 cd measured in the dark with the digital LED luxmeter from a distance of 8 cm. When Figures 3a and 3b were compared, the rate of decrease in the absorbance of the mixture containing compound **6** and DPBF at a wavelength of 410 nm was seen to be higher than the degradation of DPBF when exposed to light. The graphs of the normalized state of absorbance values at 410 nm in Figures 3a and 3b provided a better understanding of this difference (Figure 3c). As could be understood from the interpretation of the graph in Figure 3c, the BOPIM (**6**) had singlet oxygen production capacity despite being excited at a wavelength of 440 nm.

**Figure 4:**
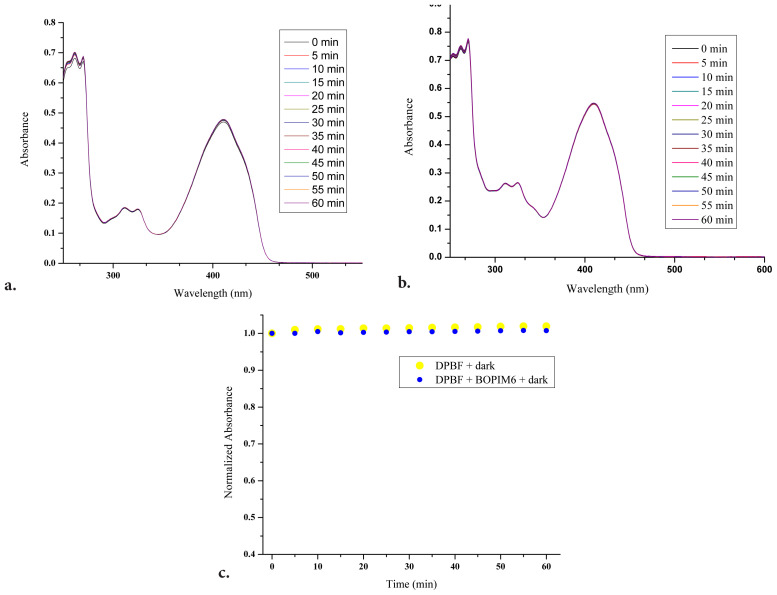
In the dark, the absorbance spectra of a) DPBF (25 μM) and b) the solution with DPBF (25 μM) and BOPIM complex (**6**) (50 μM). c) Graph of the normalized values at 410 nm in a and b with respect to time.

The necessary measurements were performed in a dark environment to examine the decomposition status of DPBF and to understand whether the BOPIM (**6**) showed a toxic effect by producing singlet oxygen. The absorbance spectra of the solution containing only DPBF (25 μM) and the other solution containing BOPIM (**6**) (50 μM) and DPBF (25 μM) in isopropyl alcohol were measured every 5 min for 60 min in the dark as well as under light of 440 nm (Figures 4a and 4b). As seen in Figure 4a, little degradation of DPBF was observed for 1 h in the dark. When Figure 4b was examined, it was seen that there were no meaningful changes in the absorbance values of DPBF in the presence of molecule **6**.

## 4. Conclusion

In the literature, structures of BOPIM cores derived from its imidazole side are commonly encountered. In this study, the derivation of a BOPIM core by pyridine was carried out with the pyrazole aromatic group (5-membered ring). The derivatives of the pyrazole molecule were shown to influence the inhibition of fungal growth in the literature. Following the synthesis and characterization of the target molecule (**6**), its photophysical properties were investigated in different solvents with different polarities. In addition, before investigating the antimicrobial photodynamic effect of the BOPIM (**6**) on microbial cell lines, its ability to generate the required singlet oxygen in PDT was investigated. It is known that DPBF decays very rapidly under light at 420 nm. To obtain the aim of this study, it was evaluated at 440 nm, a wavelength that not only excited the BOPIM (**6**) complex but also did not cause rapid degradation of DPBF. As a result of the measurements, it was revealed that the BOPIM (**6**) had a very high Stokes shift of 104 nm compared to those of many fluorescent molecules. Additionally, according to measurements regarding singlet oxygen generation, the BOPIM (**6**) has the potential to produce singlet oxygen and is only active under light at 440 nm, which has energy lower than 400 nm, at which **6** can be excited best. It is thought that these results will be pioneering in the design and synthesis of new BOPIM derivatives and in the investigation of their effects on aPDT.

## Supplementary information: Spectral data

Figure S1 ^1^H NMR spectrum of compound **3**..

Figure S2 ^13^C NMR spectrum of compound **3**.

Figure S3 TOF-HRMS spectrum of compound **3**.

Figure S4 ^1^H NMR spectrum of compound **4**.

Figure S5 ^13^C NMR spectrum of compound **4**.

Figure S6 TOF-HRMS spectrum of compound **4**.

Figure S7 ^1^H NMR spectrum of compound **5**.

Figure S8 ^13^C NMR spectrum of compound **5**.

Figure S9 TOF-HRMS spectrum of compound **5**.

Figure S10 ^1^H NMR spectrum of compound **6**.

Figure 11 ^13^C NMR spectrum of compound **6**.

Figure S12 HSQC spectrum of compound **6**.

Figure S13 TOF-HRMS spectrum of compound **6**.

## Figures and Tables

**Scheme:**
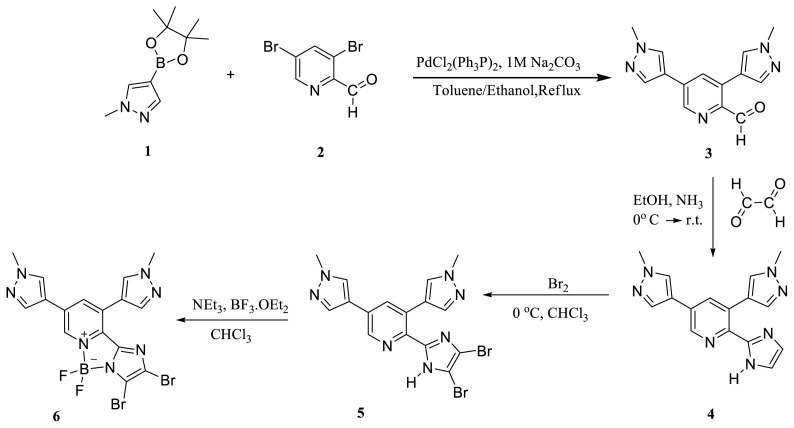
Structural view and synthesis pathway of BOPIM (**6**).

**Table:** Absorbance, λ_abs_, ɛ, λ_ems_, and Φ_em_ values of BOPIM (**6**).

Solvents	Abs.	λ_max_ (abs, nm)	ɛ × 10^4^ (M^−1^ cm^−1^)	λ_max_ (em, nm)	Φ_em_
EtOAc	0.05	403	0.180	487	0.249
CH_2_Cl_2_	0.05	406	0.184	491	0.249
THF	0.04	406	0.152	492	0.262
CH_3_CN	0.05	393	0.180	497	0.243
CH(CH_3_)_2_OH	0.04	404	0.152	490	0.259
CH_3_OH	0.04	397	0.176	493	0.226
