# Supplementary figures and images for: Synthesis and singlet oxygen generation of boron-2-(4,5-dibromo-1H-imidazole-2-yl)-3,5-dipyrazolopyridine complex for antimicrobial photodynamic therapy
Source: Turk J Chem. 2023 Oct 11;47(6):1452–8. doi: 10.55730/1300-0527.3627 (PMC10965182; doi:10.55730/1300-0527.3627)

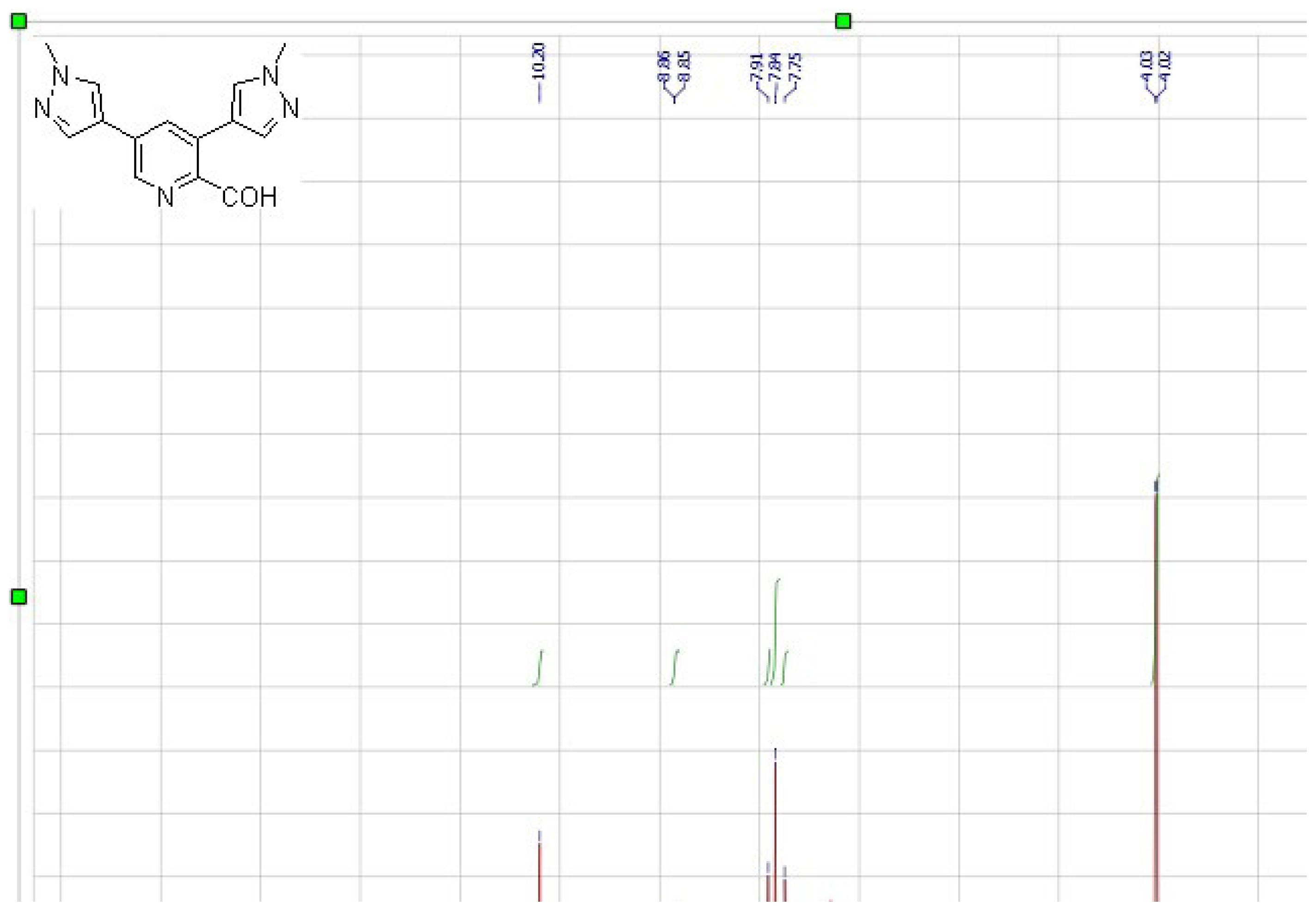

Supplement: Figure S1 — 1H NMR spectrum of compound 3.. [file tjc-47-06-1452s1.tif]

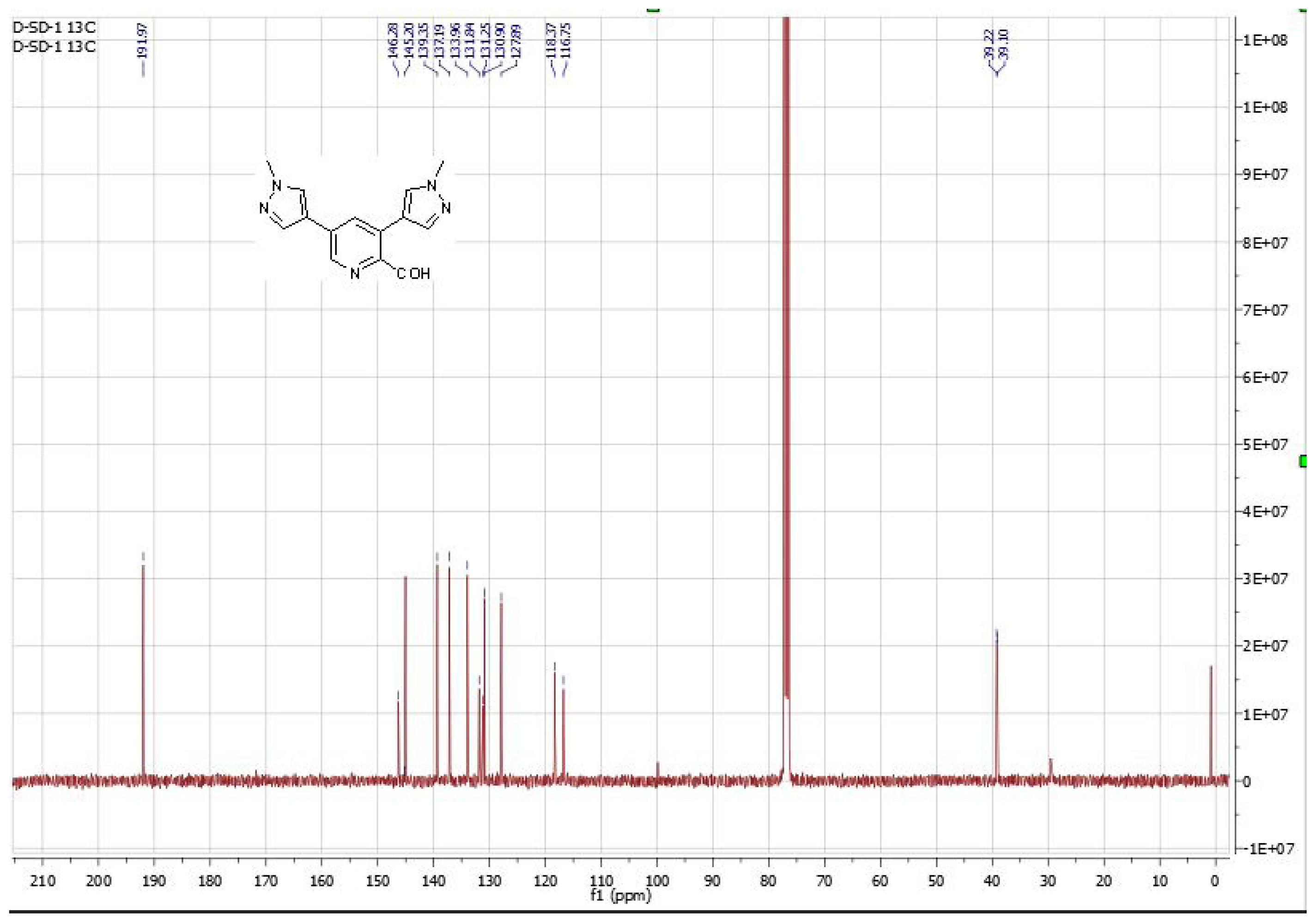

Supplement: Figure S2 — 13C NMR spectrum of compound 3. [file tjc-47-06-1452s2.tif]

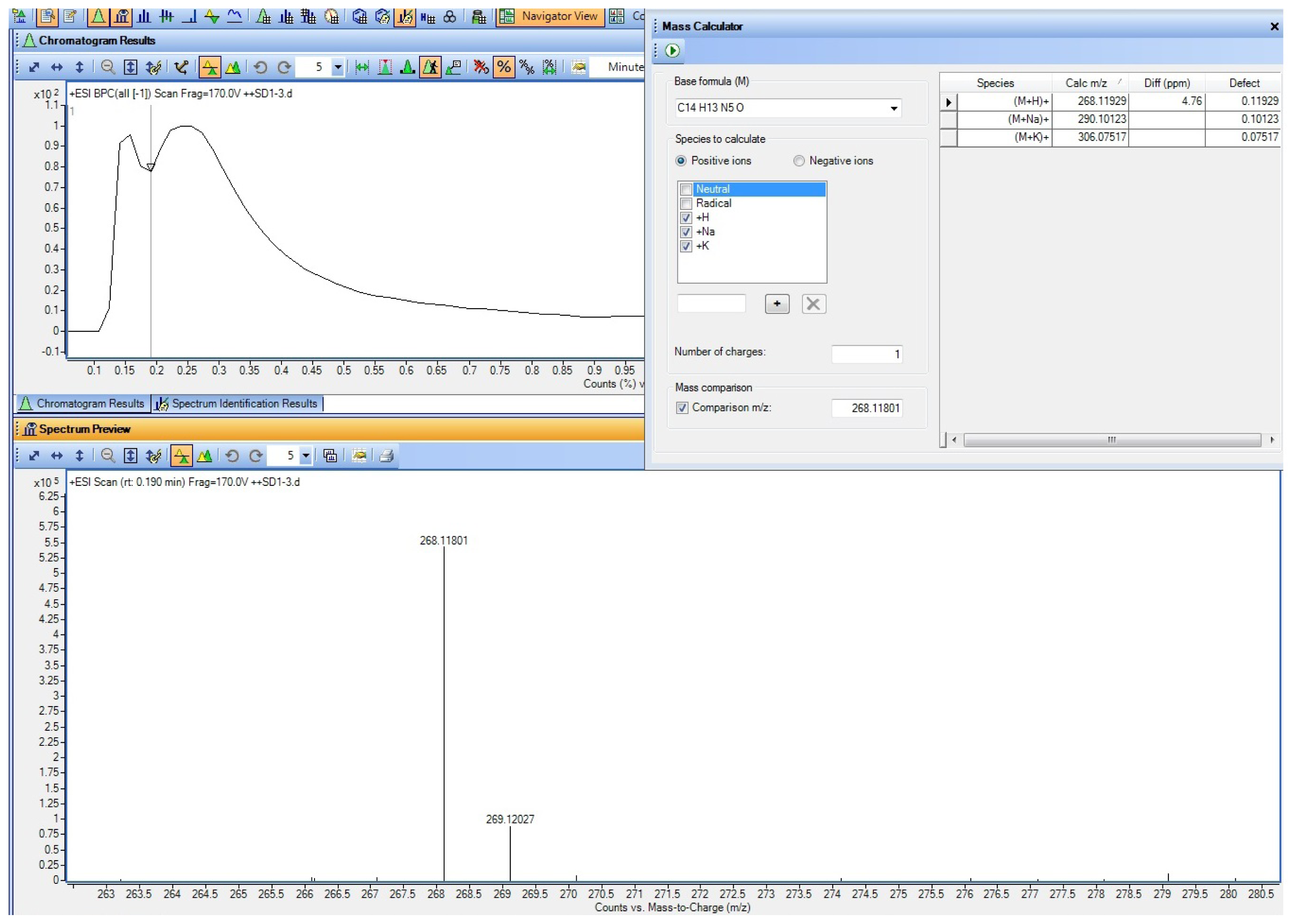

Supplement: Figure S3 — TOF-HRMS spectrum of compound 3. [file tjc-47-06-1452s3.tif]

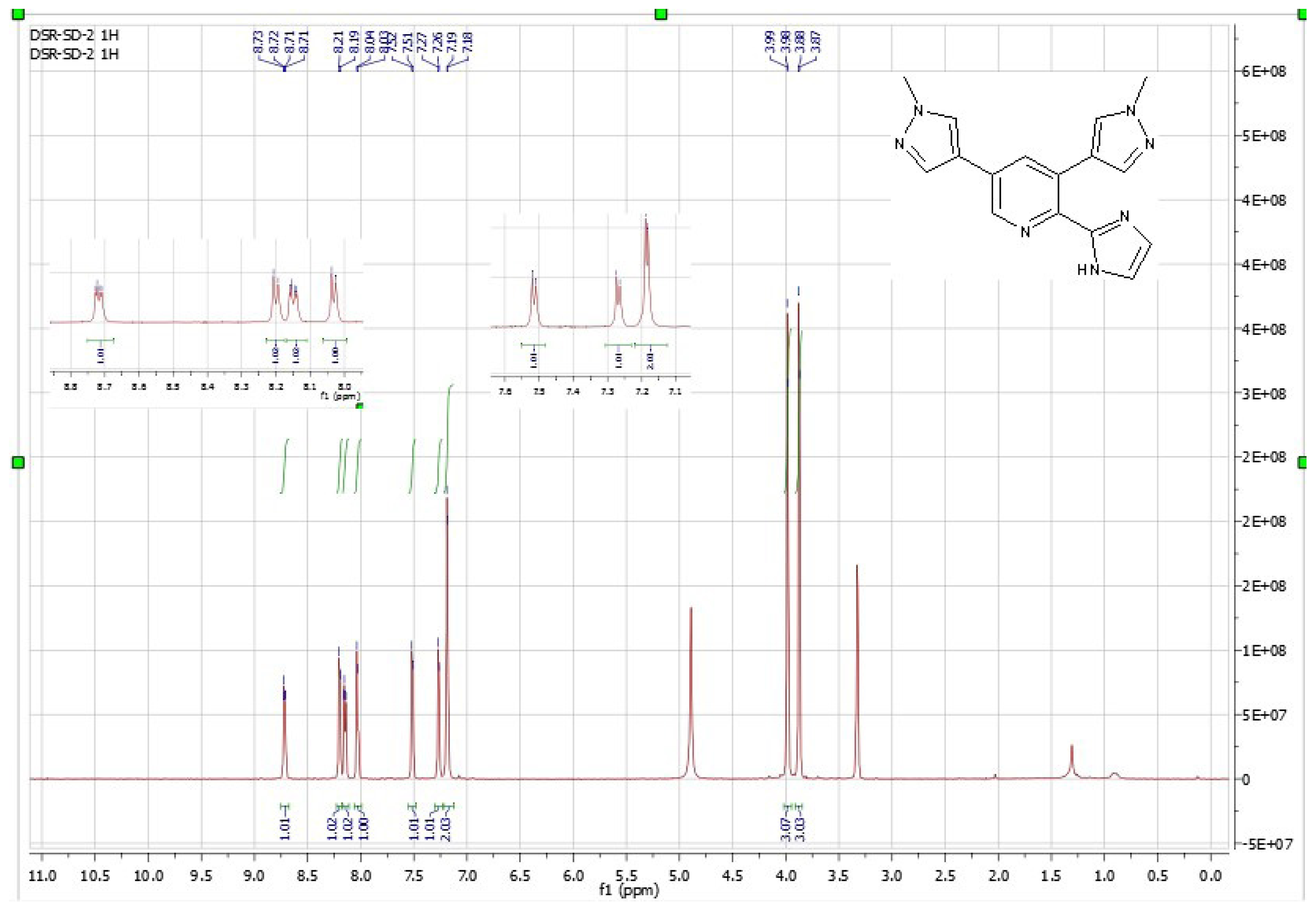

Supplement: Figure S4 — 1H NMR spectrum of compound 4. [file tjc-47-06-1452s4.tif]

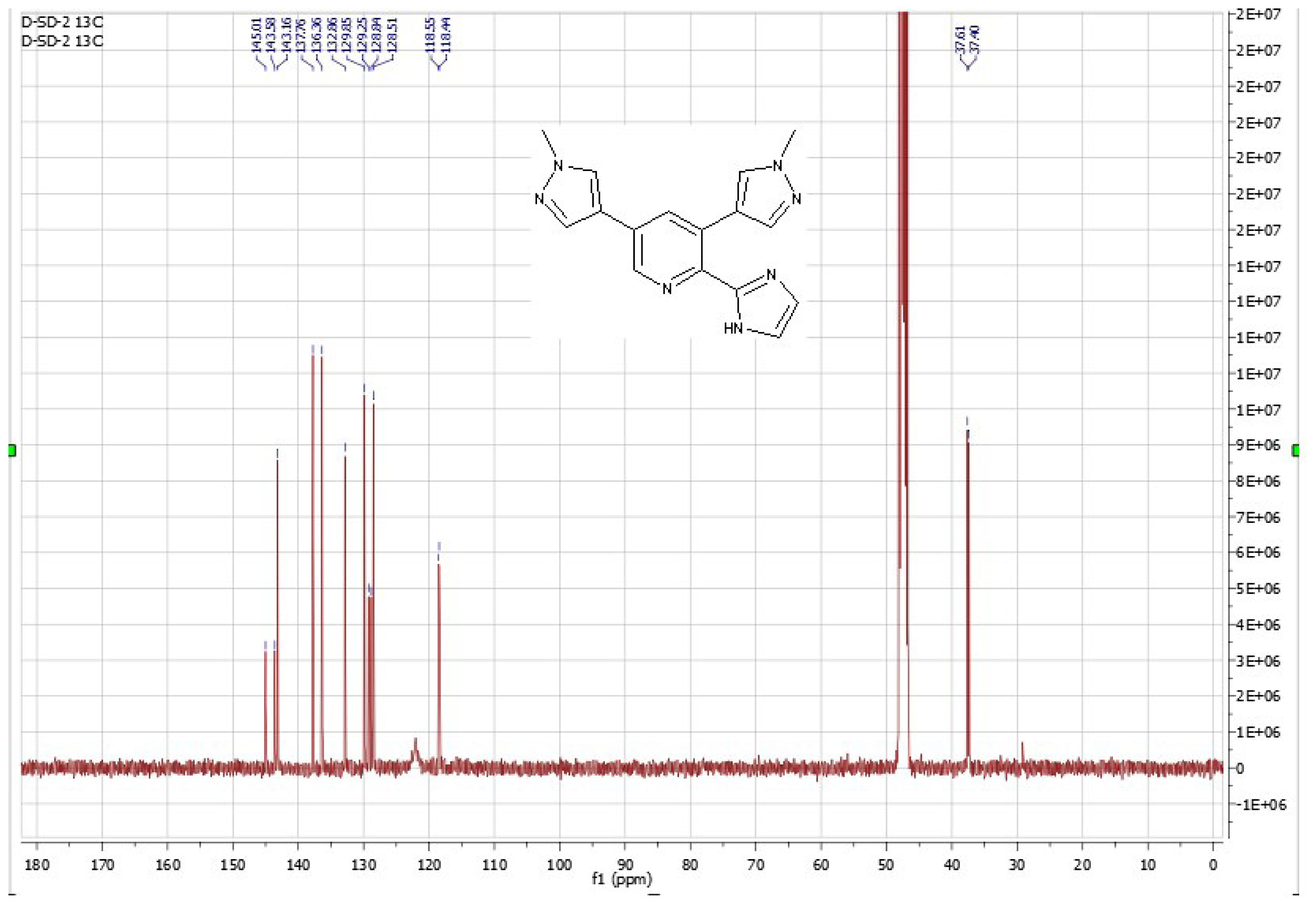

Supplement: Figure S5 — 13C NMR spectrum of compound 4. [file tjc-47-06-1452s5.tif]

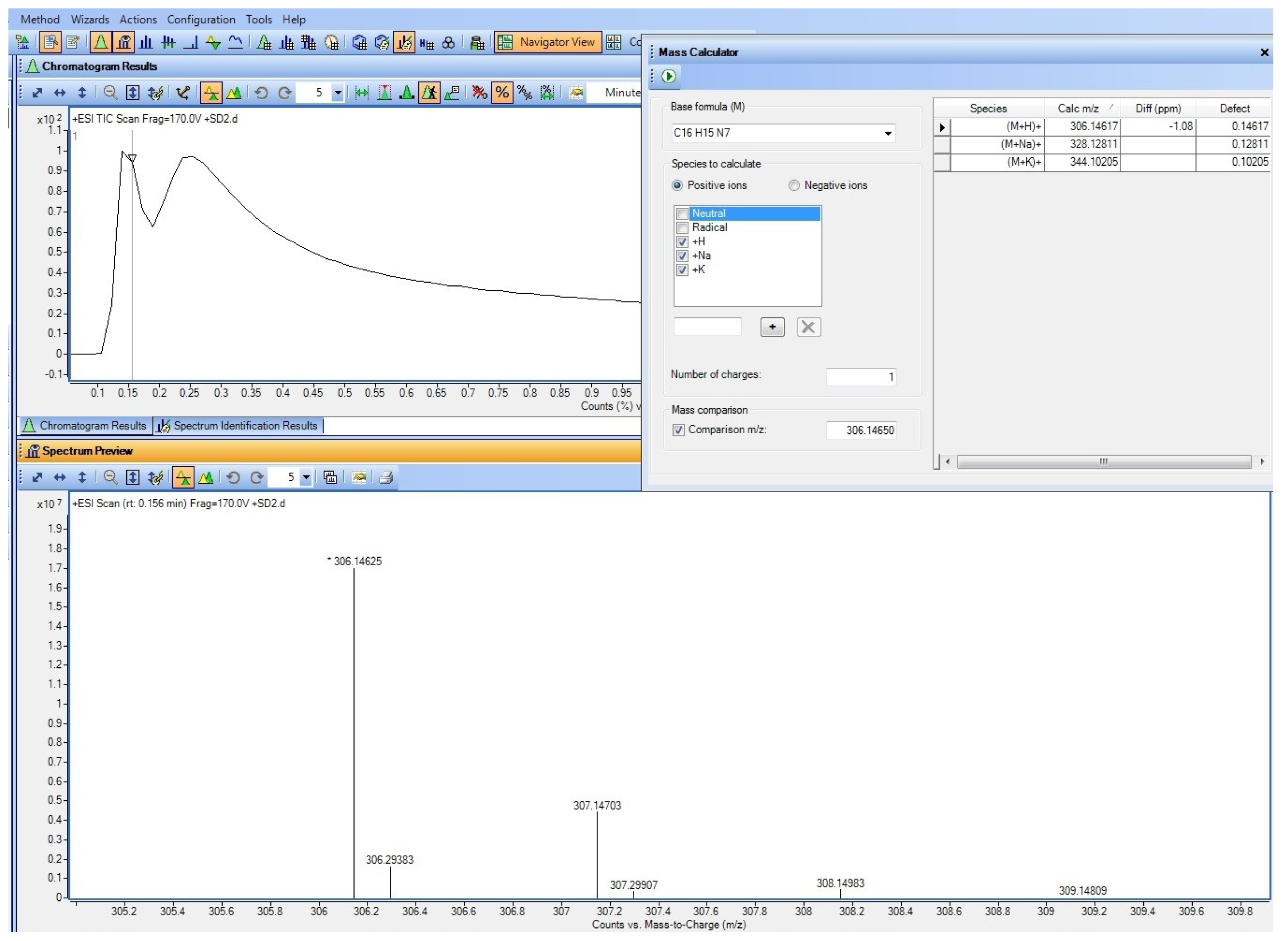

Supplement: Figure S6 — TOF-HRMS spectrum of compound 4. [file tjc-47-06-1452s6.tif]

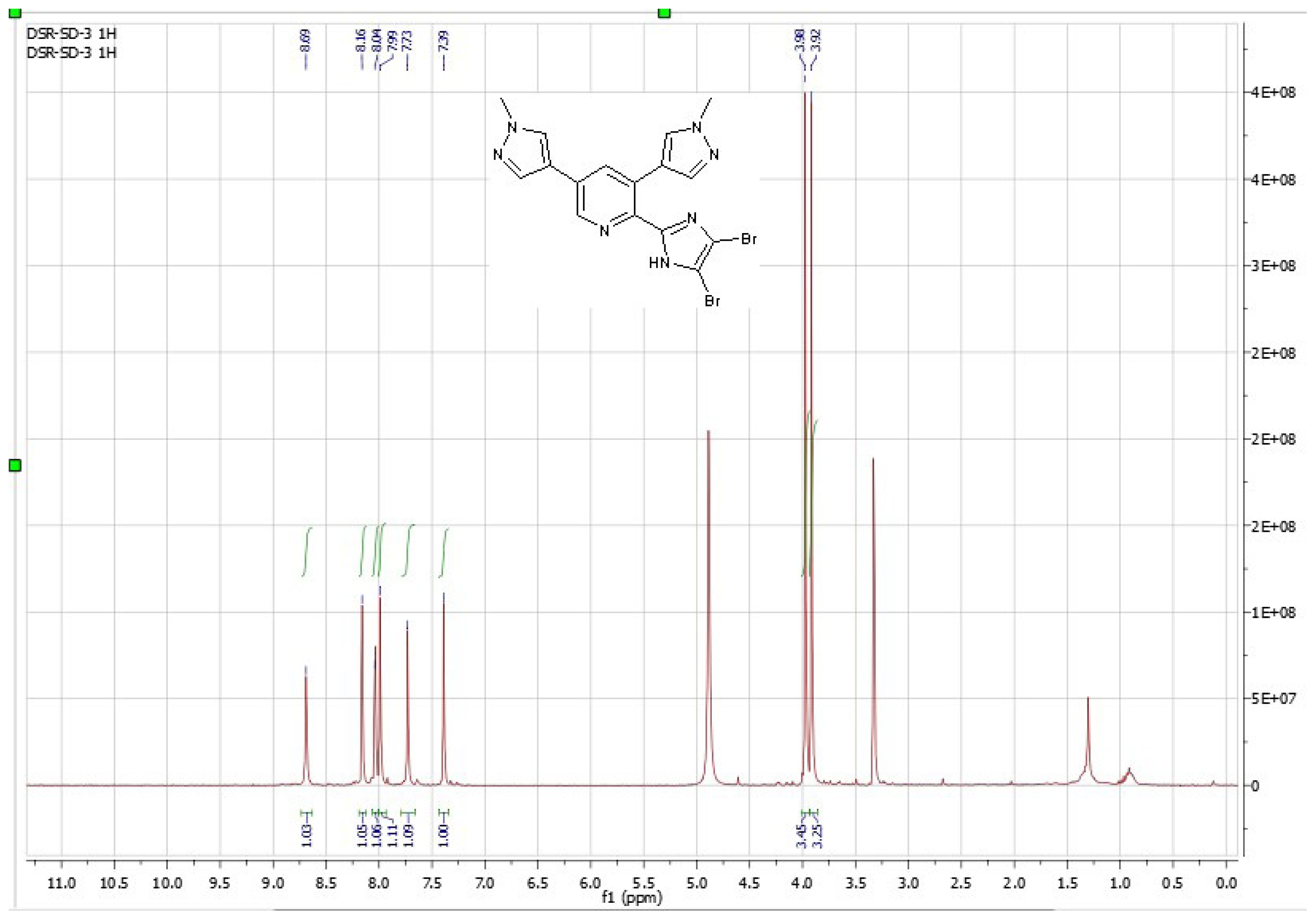

Supplement: Figure S7 — 1H NMR spectrum of compound 5. [file tjc-47-06-1452s7.tif]

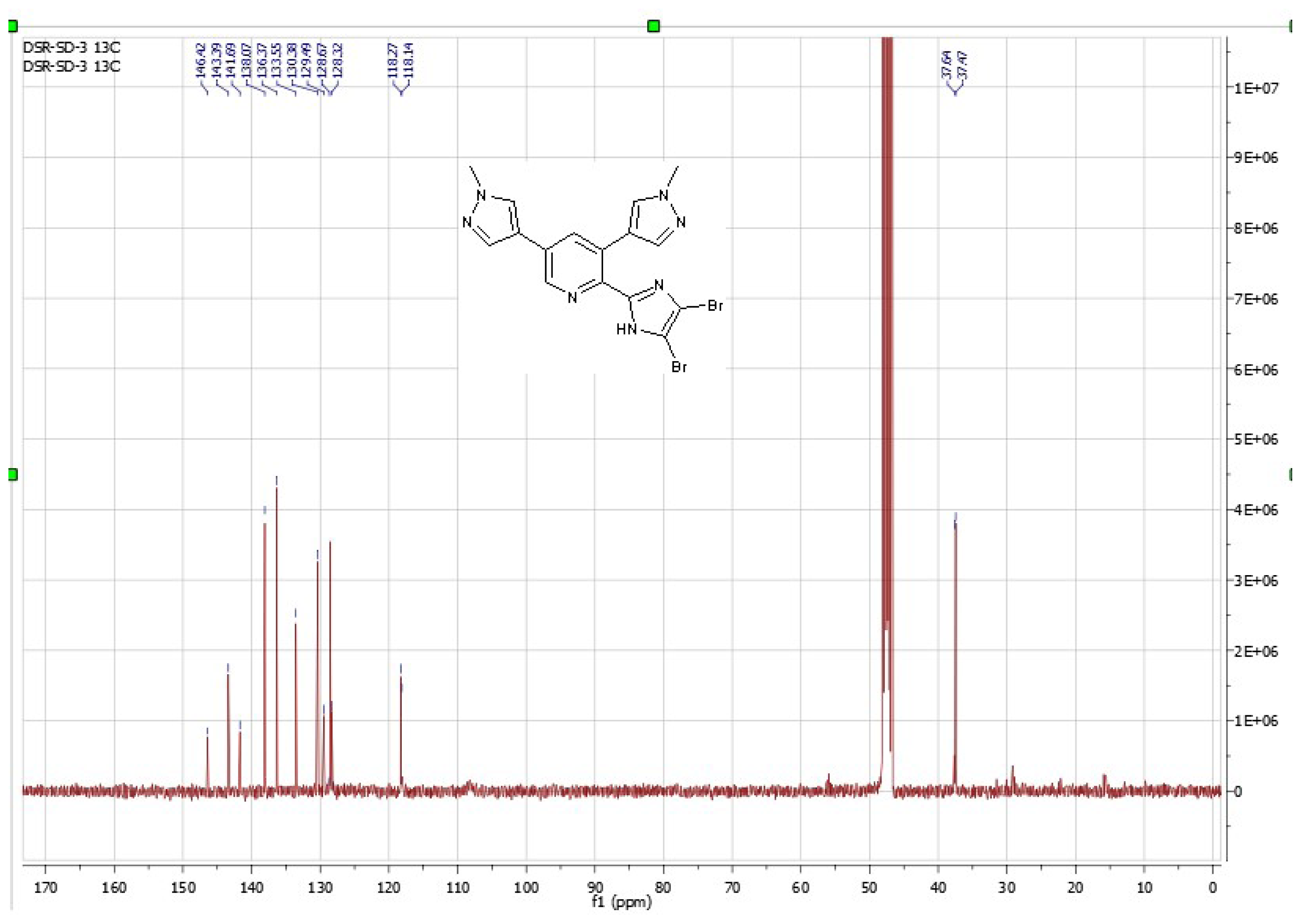

Supplement: Figure S8 — 13C NMR spectrum of compound 5. [file tjc-47-06-1452s8.tif]

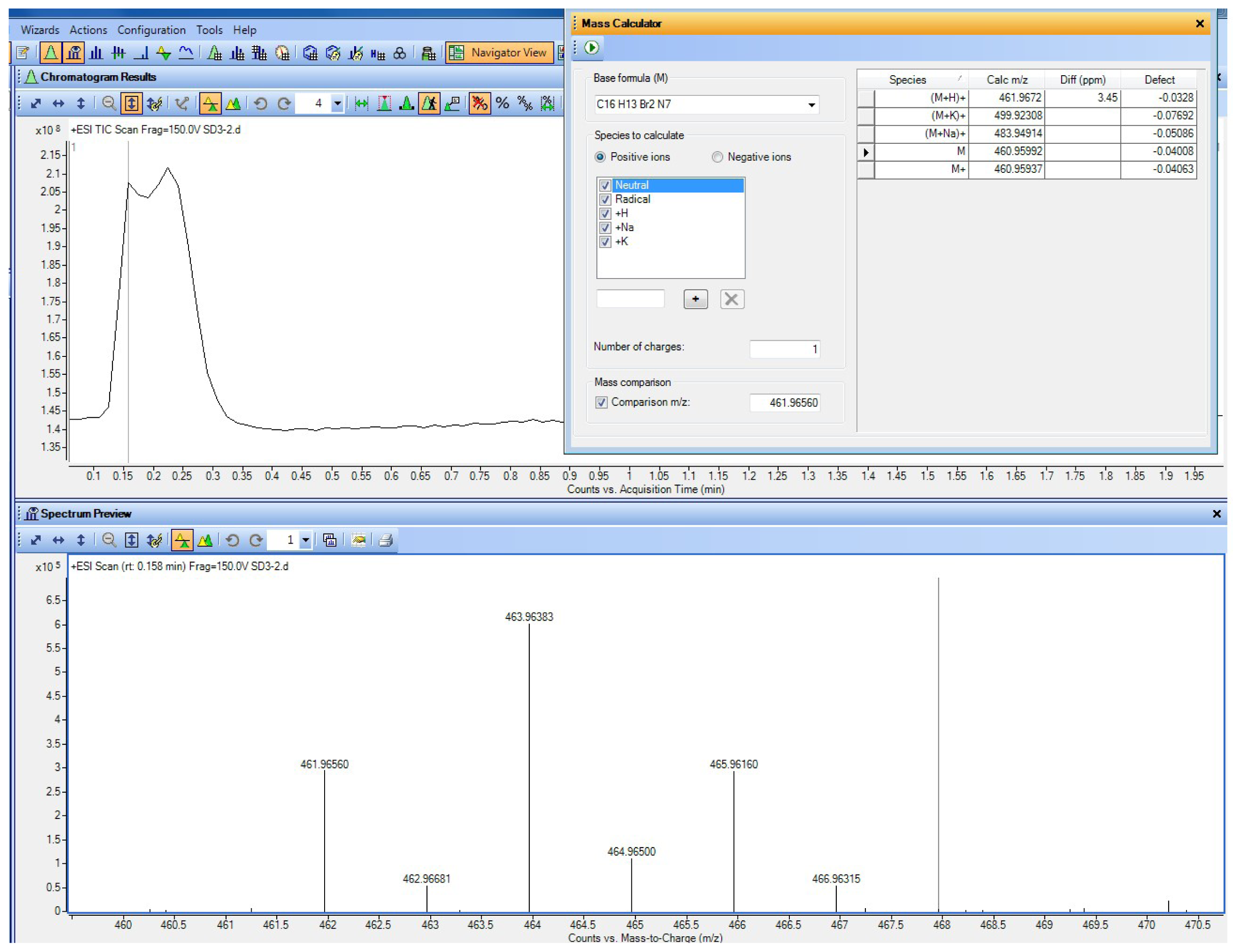

Supplement: Figure S9 — TOF-HRMS spectrum of compound 5. [file tjc-47-06-1452s9.tif]

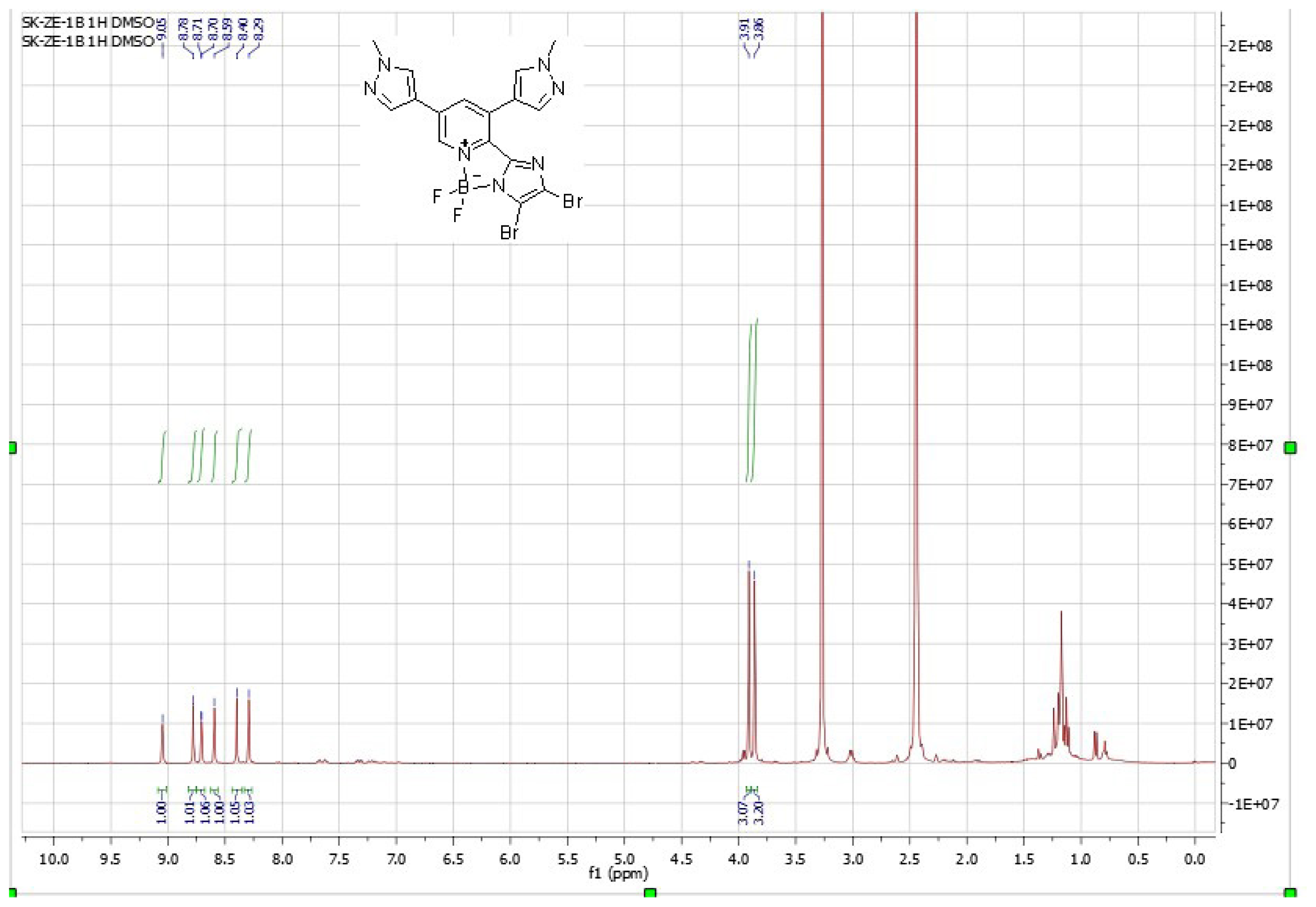

Supplement: Figure S10 — 1H NMR spectrum of compound 6. [file tjc-47-06-1452s10.tif]

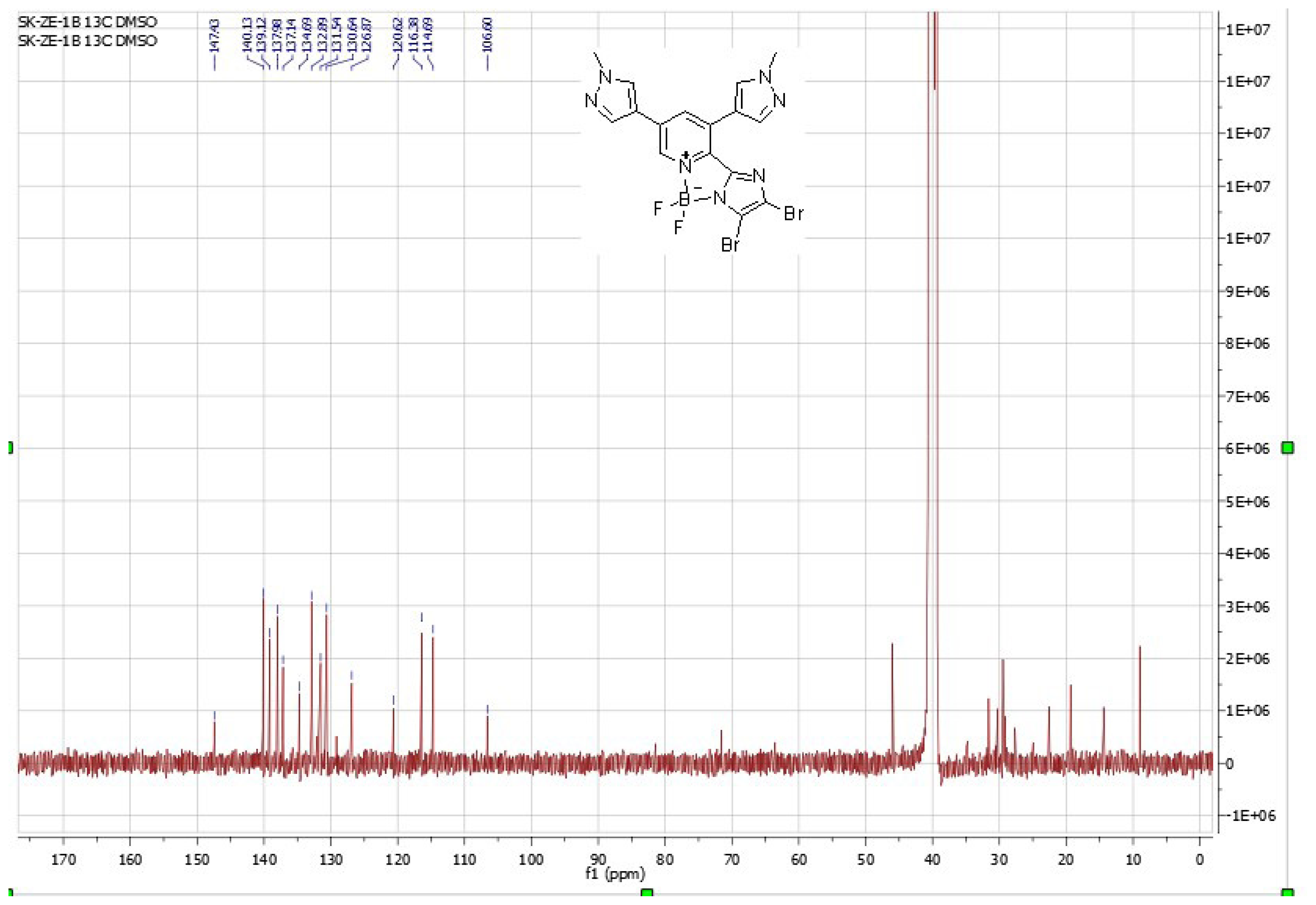

Supplement: Figure 11 — 13C NMR spectrum of compound 6. [file tjc-47-06-1452s11.tif]

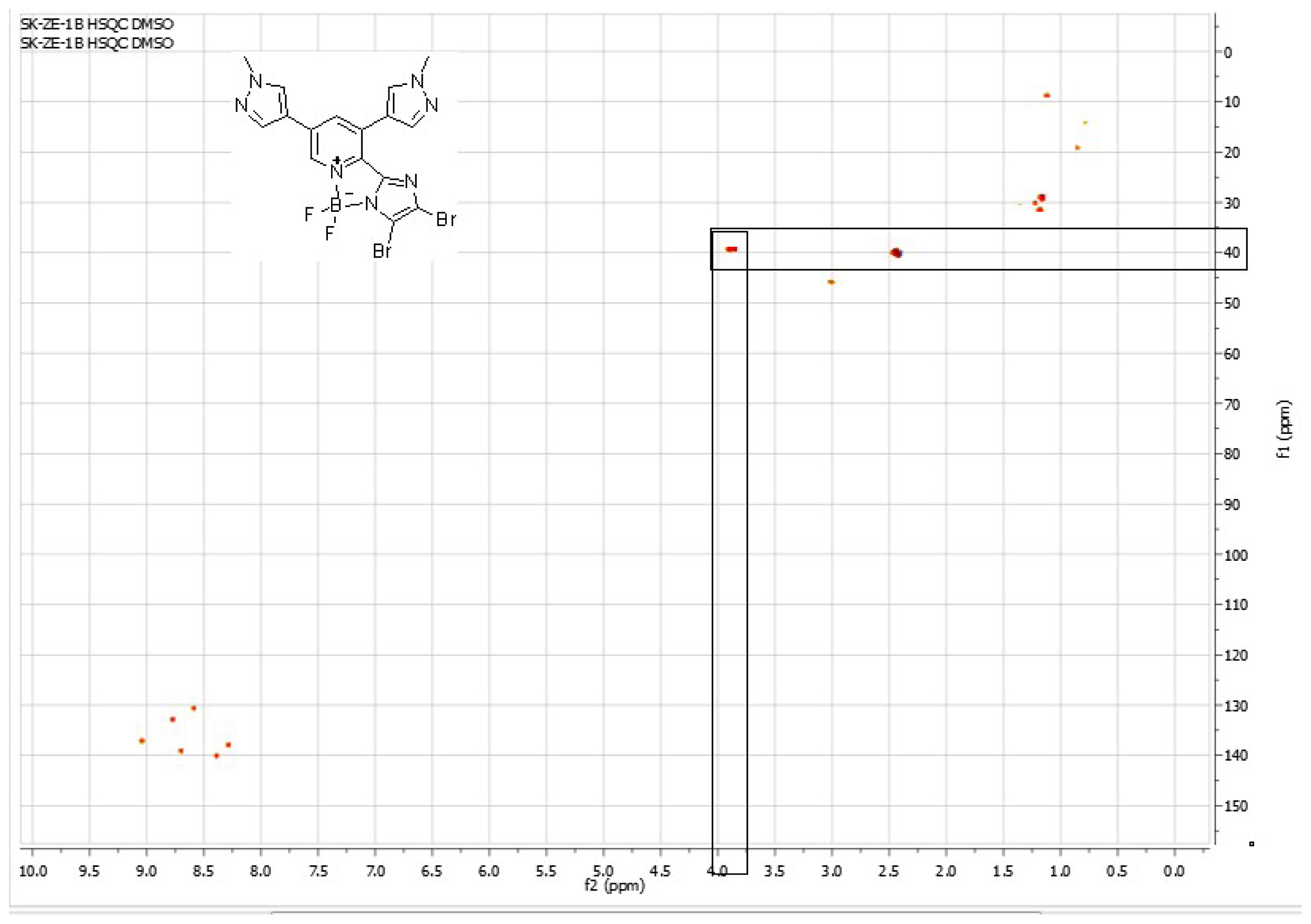

Supplement: Figure S12 — HSQC spectrum of compound 6. [file tjc-47-06-1452s12.tif]

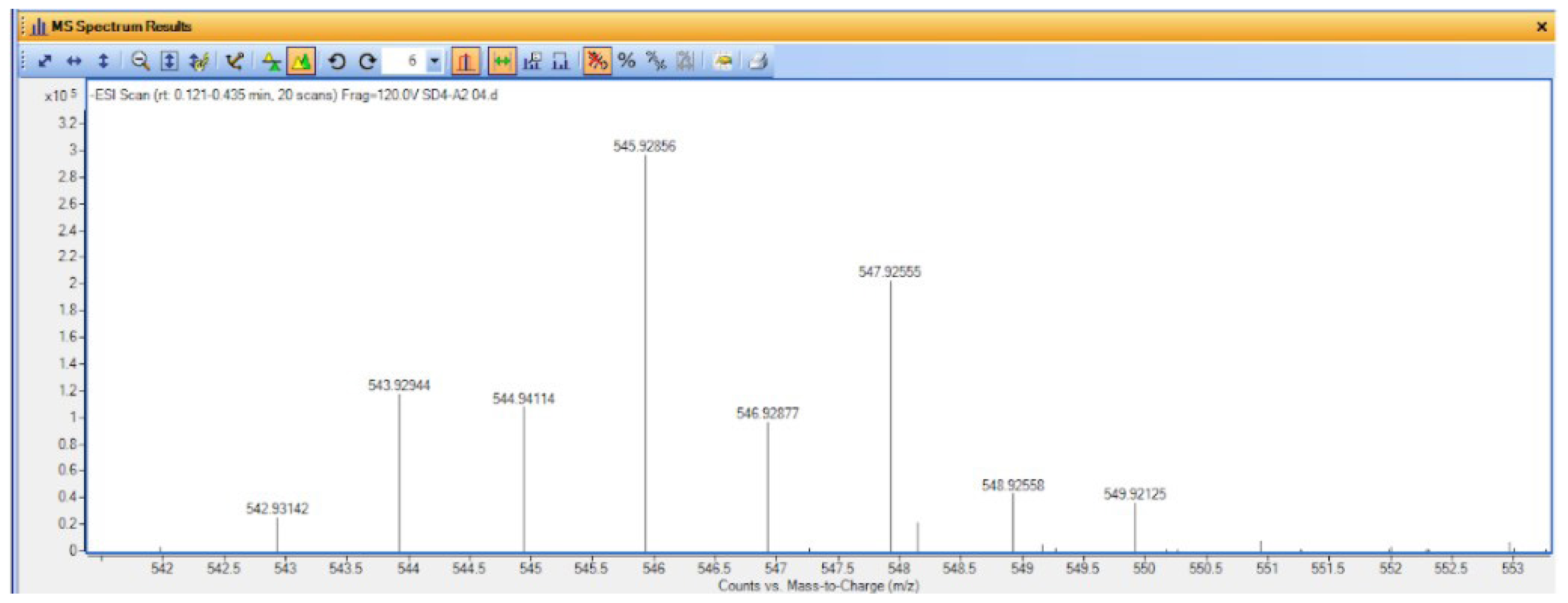

Supplement: Figure S13 — TOF-HRMS spectrum of compound 6. [file tjc-47-06-1452s13.tif]
